# Paediatric Hemophagocytic Lymphohistiocytosis: A Case Series With a Diverse Spectrum From a Resource-Limited Setting

**DOI:** 10.7759/cureus.45140

**Published:** 2023-09-12

**Authors:** Sanghamitra Ray, Manish Kumar, Nidhi Mahajan, Arti Khatri

**Affiliations:** 1 Department of Pediatrics, Vardhaman Mahavir Medical College (VMMC) and Safdarjung Hospital, Delhi, IND; 2 Department of Pediatrics, Chacha Nehru Bal Chikitsalaya, Delhi, IND; 3 Department of Pathology, Chacha Nehru Bal Chikitsalaya, Delhi, IND

**Keywords:** sars-cov-2 associated hlh, idiopathic hlh, leptospirosis associated hlh, dengue associated hlh, infection associated hlh, secondary hlh, pediatric, hemophagocytic lymphohistiocytosis

## Abstract

Hemophagocytic lymphohistiocytosis (HLH) is a hyperinflammatory syndrome characterized by cytokine storms leading to multi-organ dysfunction and is a highly fatal disease. Infectious diseases are the most common cause of secondary HLH. A wide variety of infections can lead to secondary HLH.

In this case series, we report five cases of HLH which had different therapeutic approaches and varied clinical courses, with one of them diagnosed as a rare entity of coronavirus disease 2019 (COVID-19)-associated HLH of multisystem inflammatory syndrome in children (MISC) spectrum, one case each of idiopathic HLH, staphylococcal infection-associated secondary HLH, leptospirosis with secondary HLH and dengue-associated HLH. The case of idiopathic HLH required initiation of immunosuppressive therapy but had a fatal outcome while others were treated successfully with antibiotics, steroids, intravenous immunoglobulin and supportive therapy.

Our case series highlights the importance of evaluating for all possible infective causes thoroughly in HLH. Most patients can be managed without chemotherapy by treating the secondary causes of HLH, including common tropical infections and severe acute respiratory syndrome coronavirus 2 (SARS-CoV-2) infection.

## Introduction

Hemophagocytic lymphohistiocytosis (HLH) is a fatal disease in which there is uncontrolled activation of the immune system secondary to genetic predisposition, infections, malignancy or immune disorders. It is characterised by fever, pancytopenia, and splenomegaly along with hemophagocytosis in bone marrow, liver or lymph nodes. HLH is defined by certain clinical and laboratory criteria which includes at least five of the following eight findings: fever ≥38.5°C, splenomegaly, cytopenia, with at least two of the following: hemoglobin <9 g/dL, platelets <100,000/microL; absolute neutrophil count <1000/microL, hypertriglyceridemia (fasting triglycerides >265 mg/dL) and/or hypofibrinogenemia (fibrinogen <150 mg/dL), and hemophagocytosis in bone marrow. HLH can be primary or secondary in origin. In this case series, we have reported five interesting cases of HLH which had different causations, varied clinical courses and therapeutic approaches.

## Case presentation

Case 1

**Figure 1:**
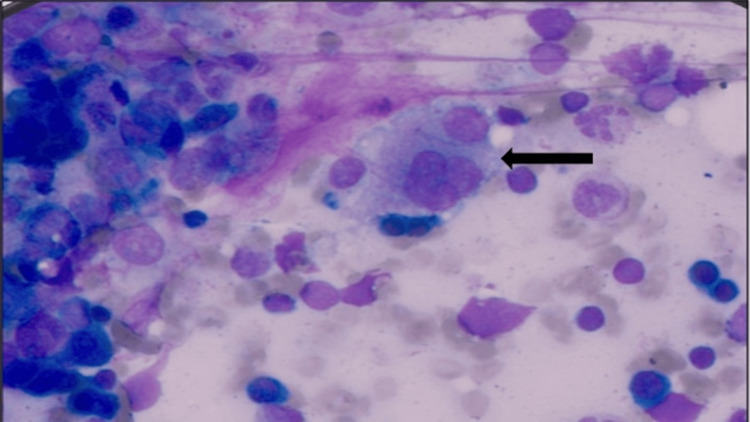
Bone marrow aspiration of case 1 showing histiocytes with engulfed cells inside (black arrow)

An eight-month-old male child had a fever of 15 days. The child was pale with hepatosplenomegaly. Initial investigation revealed pancytopenia with neutropenia (Table 1). Liver function test showed transaminitis. Lactate dehydrogenase (LDH), C-reactive protein (CRP), triglyceride, and ferritin levels were high (Table 2). Multiple urine and blood cultures including fungal workup and congenital infections workup were non-yielding. Bone marrow showed an increase in the number of histiocytes with many showing hemophagocytosis (Figure 1). As per HLH 2004 criteria, the diagnosis of HLH was established and he was started on antibiotic therapy. As the patient did not show any improvement he was started on chemotherapy with steroid, vincristine and cyclosporine. Whole exome sequencing was done but it did not reveal any pathogenic or any likely pathogenic genetic mutations. Familial HLH mutations were also negative but the cause of secondary HLH could not be identified. The child continued on intensive chemotherapy with dexamethasone, etoposide and cyclosporine for eight weeks but his clinical condition did not improve. Maintenance chemotherapy continued and the patient was given the option of bone marrow transplantation but could not opt due to financial constraints. Despite chemotherapy and supportive care, the patient succumbed to his illness at the 12th week of chemotherapy.

**Table 1:** Clinical details and hematological parameters of the cases

	Case 1		Case 2		Case 3		Case 4		Case 5
Age/sex	8 months/male		26 months/female		8 years/male		5.5 years/female		10 years/male
Presenting complaints	Fever for 15 days		Fever and loose stool for 15 days		Fever for 20 days		Fever for 1 month		Fever for 14 days
Significant clinical findings	Pallor, hepato-splenomegaly		Cervical lymphadenopathy, hepatosplenomegaly		Pallor, unilateral parotid swelling, hepato-splenomegaly		Pallor, hepato-splenomegaly		Rash, Hepatomegaly
Hematological parameters at admission									
Hemoglobin gm/dl		3.7		5.3		7.7		8.3	6.9
Total leucocyte count (per mL)		4000		37000		1300		1400	4200
Absolute neutrophil count (per mL)		1080		11840		195		238	1200
Platelet count (per mL)		47000		100000		60000		20000	30000
Reticulocyte count		0.3%		0.8%		1%		0.5%	0.8%
Bone marrow aspirate findings		Increased number of histiocytes and many of them showing erythrophagocytosis		Similar findings as case 1		Similar findings as case 1		No significant finding	Similar findings as case 1

**Table 2:** Biochemical parameters of cases at admission ALT: alanine aminotransferase, AST: aspartate aminotransferase, ALP: alkaline phosphatase, LDH: lactate dehydrogenase

	Urea/creatinine (mg/dl)	S Bilirubin (mg/dl)/ALT/AST/ALP (U/L)	LDH (IU/L)	Serum ferritin mg/dl	Serum triglyceride mg/dl	Serum albumin gm/dl	Serum sodium Meq/L
Case 1	33/0.2	2.91/231/84/126	2150	2500	599	2.03	128
Case 2	20/0.4	3.2/189/175/1220	1200	4500	518	2.6	126
Case 3	18/0.6	1.66/122/78/780	377	1728	640	2.11	125
Case 4	27/0.24	0.8/42/36	490	2044	156	4.04	129
Case 5	30/0.8	1.0/166/100/600	1180	2800	344	2.4	124

Case 2

**Figure 2:**
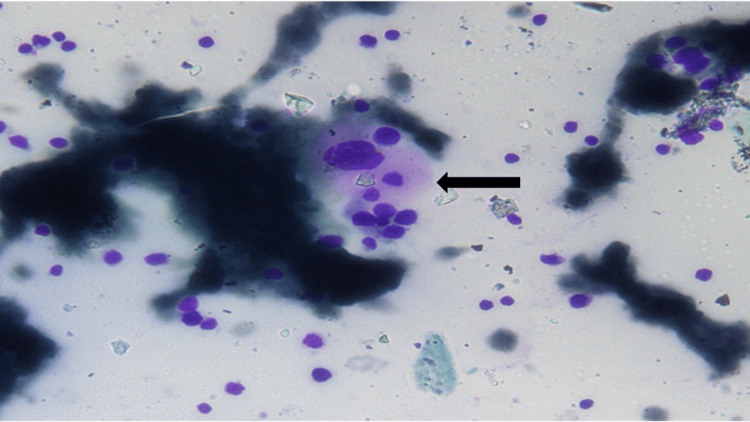
Bone marrow aspirate smear showing histiocyte with phagocytic activity in a hemorrhagic background in case 2 (black arrow) Giemsa stain, 40X

A 26-month-old female child presented with fever and loose stool for 10 days and had cervical lymphadenopathy and hepato-splenomegaly. Initial investigations revealed bicytopenia with leucocytosis and hematological and biochemical features of HLH. (Tables 1, 2). Based on these investigations the child was diagnosed as HLH. We started the child on ceftriaxone and she also received red cell transfusion. After five days as the child did not show improvement antibiotics were upgraded. Fine needle aspiration cytology of lymph nodes was suggestive of reactive lymphadenopathy. Bone marrow showed an increased number of histiocytes with active hemophagocytosis (Figure 2). Coronavirus disease 2019 (COVID-19) reverse transcription polymerase chain reaction (RT-PCR) was non-reactive but as the child had a history of contact with a COVID-19 case in the family we had the COVID-19 antibody test done which showed highly elevated immunoglobulin M (IgM) (33.05 AU/ml). Therefore, the child was diagnosed with COVID-19-associated HLH. Autoimmune workup and immunoglobulin profile were normal. The child improved with steroid therapy and was asymptomatic for three months and subsequently lost to follow-up. 

Case 3

An eight-year-old male child presented with fever for 20 days and on examination had pallor, unilateral parotid swelling and hepato-splenomegaly. On routine investigation, the child had pancytopenia with neutropenia (Table 1). Liver enzymes were deranged, and albumin and sodium were low. The child also had high CRP, LDH and ferritin levels (Table 2). Blood cultures showed growth of methicillin-resistant Staphylococcus aureus (MRSA). Bone marrow aspirate showed increased histiocytes with hemophagocytosis. Diagnosis of HLH with staphylococcal infection with febrile neutropenia was made. Antibiotics were upgraded to vancomycin according to the culture sensitivity. Immunoglobulin profile was normal and workup for autoimmune profile was non-yielding. Genetic analysis could not be done due to non-affordability. Gradually the patient improved and became afebrile after seven days of upgrading the antibiotics. He was discharged and is now under regular follow-up. He did not show any features of relapse until the last follow-up at seven months.

Case 4

A five-year-old girl was admitted with us with complaints of fever for the last 20 days. She was admitted to a private hospital where she was transfused with packed red cells at a hemoglobin of 3.2gm% (Table 1). There was no other significant systemic symptom. On examination she was pale with hepatosplenomegaly. Hemogram at admission showed pancytopenia and the initial provisional diagnosis was kept as sepsis with pancytopenia with febrile neutropenia. Peripheral smear examination did not reveal blasts. The child was started on piperacillin and amikacin and sepsis workup was sent. Workup for tropical infections such as malaria, enteric fever, and dengue was negative. Blood culture did not reveal any growth. As fever and pancytopenia persisted, antibiotics were upgraded to meropenem and vancomycin. On day five an antifungal was added as per standard protocol for febrile neutropenia. Detailed fungal workup including fungal culture was sent. The possibility of HLH was kept and investigations were sent which showed high ferritin and triglyceride while prothrombin time (PT), activated partial thromboplastin time (aPPT), and international normalised ratio (INR) were normal. The child had hyponatremia (Table 2). According to the HLH 2009 criteria the child was diagnosed as HLH. On further workup for atypical organisms, Leptospira and rickettsial serology was sent and Leptospira IgM was positive, making the diagnosis leptospirosis with secondary HLH. Genetic analysis for HLH mutations could not be done due to financial constraints. An autoimmune antibody panel was negative. Chemotherapy was not needed as the child improved on antibiotic therapy. Antifungals were stopped as there was no evidence of fungal infection. On the 10th day of admission, the child showed improvement in neutropenia, her fever gradually declined and she could be discharged after completion of the antibiotic course. The child was asymptomatic until six months of follow-up.

Case 5

**Figure 3:**
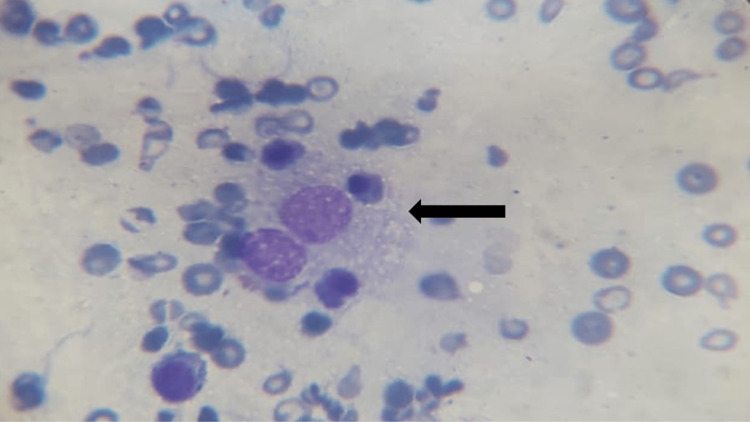
Bone marrow aspirate smear of case 5 showing histiocyte with active phagocytosis (black arrow) Giemsa stain, 40X

A 10-year-old male child presented to the Outpatient Department with complaints of fever for four days, gum bleeding and macular erythematous rash. He had bicytopenia and hepatomegaly (Table 1). Liver functions were deranged. Dengue IgM was positive, and IgG was negative. He was being managed as dengue fever but continued to have a fever for 14 days after admission despite having a negative septic workup. Repeat examination showed enlarging hepatosplenomegaly, high ferritin, and hyponatremia (Table 2). Bone marrow was also suggestive (Figure 3) and he was diagnosed as dengue-associated secondary HLH. He was treated with steroids and supportive care and had recovered fully with treatment. Second-line immunosuppressive treatment was not required for the child. 

To establish the cause of HLH, detailed microbiological investigations were done in all five cases to rule out secondary causes of infections. Blood culture, urine culture, malaria (peripheral smear for malaria and rapid malarial antigen test), serum widal, dengue, rickettsial and leptospiral serology, test for human immunodeficiency virus, test for COVID-19 (rapid antigen test and RT-PCR) and COVID-19 antibody level. Case 1 was diagnosed as idiopathic secondary HLH, case 2 as COVID-19-associated HLH of multisystem inflammatory syndrome in children (MISC) spectrum, case 3 as staphylococcal infection-associated secondary HLH, case 4 as leptospirosis with secondary HLH, and case 5 as dengue hemorrhagic fever-associated secondary HLH (Table 3). 

**Table 3:** Final diagnosis, treatment and outcome of the cases HLH: hemophagocytic lymphohistiocytosis, MISC: multisystem inflammatory syndrome in children, MRSA: methicillin-resistant Staphylococcus aureus, Ig: immunoglobulin

Patient ID	Final diagnosis	Diagnostic test	Treatment	Outcome
Case 1	Idiopathic secondary HLH	normal whole exome sequencing	Chemotherapeutic drugs according to the HLH 2004 protocol.	Death
Case 2	COVID-19-associated HLH of MISC spectrum	High COVID-19 antibody titre (IgM 33.05 AU/ml)	Immunoglobulin and steroids	Recovered
Case 3	Staphylococcal infection-associated secondary HLH	Blood culture growth of MRSA	Antibiotic (vancomycin)	Recovered
Case 4	Leptospirosis-associated secondary HLH	Leptospira IgM positive (microscopic agglutination test)	Antibiotic (azithromycin) and steroid	Recovered
Case 5	Dengue hemorrhagic fever-associated HLH	Dengue IgM positive, IgG negative	Steroid and symptomatic treatment	Recovered

Idiopathic secondary HLH was treated with chemotherapeutic drugs according to the HLH 2004 protocol which consisted of an intensive phase of eight weeks and a continuation phase of the ninth to 24th weeks. Drugs included as per protocol were dexamethasone, etoposide and cyclosporine. COVID-19-associated HLH of MISC spectrum was treated with methylprednisolone 2 mg/kg/day for three days, followed by oral prednisolone 2mg/kg/day in tapering doses over seven to 10 days. Echocardiography was normal at diagnosis and one-month follow-up. Staphylococcal infection-associated secondary HLH was treated with vancomycin whereas leptospirosis with secondary HLH was successfully treated with azithromycin. Dengue hemorrhagic fever-associated HLH was treated with dengue treatment protocol and steroid.

## Discussion

HLH is classically diagnosed using HLH-94 protocol [1,2] and subsequently by the modified HLH-2004 protocol [3]. There are many case series on pediatric HLH from India [4-6] and these studies showed that infections are always a major trigger in secondary HLH. Ramachandran et al. studied 43 children with HLH and found infection as a causative factor for HLH in 42% of the cases [5]. Although the entity of severe acute respiratory syndrome coronavirus 2 (SARS-CoV-2)-associated HLH is not very common as of now, it is evident that a systemic hyper-inflammatory state plays an important role in multi-organ dysfunction and Kawasaki disease-like presentation [7]. There are a few recent case reports in adults where SARS-CoV-2 was attributed as a cause of HLH [8,9]. There are a few case reports in the pediatric population too where SARS-CoV-2 was associated with HLH [10]. The diagnosis of MISC requires presence of fever, inflammation, and multisystem (more than two) involvement, current or recent SARS‐CoV‐2 infection and no alternative plausible diagnosis and our case 2 fulfilled all the criteria. Although zoonotic diseases are common causes of HLH, leptospirosis-associated HLH has been rarely reported in the literature and pediatric cases are even rare [11]. One of the hypotheses behind the pathogenesis of leptospirosis-associated HLH is that Leptospira might induce a tumor necrosis factor α (TNFα)-associated apoptosis of macrophages, along with interleukin over-production leading to cytokine storm and multiorgan failure [12], even though the exact mechanism is still unclear yet. Jevtic et al. described a 13-year-old child with leptospirosis-associated HLH who had a favourable outcome with antibiotic and steroid [13]. Staphylococcus as a cause of HLH is rare in literature. Staphylococcus was implicated as a cause of secondary HLH in two cases in a pediatric case series including 52 children with HLH [14]. We could find a case report of an adult female with HLH secondary to methicillin-resistant Staphylococcus epidermidis (MRSE)-related left-sided infectious endocarditis. In our index case as exome sequencing was not done it is difficult to deny the presence of any heterozygous mutation of the familial HLH gene. Another study from India showed that out of 28 sepsis-associated HLH cases, there was only one case (3.6%) of sepsis-associated HLH due to MRSA [15]. Children with severe dengue are also at a higher risk of developing secondary HLH, which contributes to the high mortality. There are several case reports of HLH as a secondary complication of dengue infection in the pediatric population where no other underlying risk factors were documented [16,17]. Dengue-associated HLH must be suspected in proven dengue cases where there is persistent fever beyond one week along with high ferritin and deteriorating cytopenia as early recognition of HLH can reduce the mortality in such cases.

## Conclusions

HLH is an underdiagnosed entity as many times it is difficult to differentiate it from fulminant sepsis. HLH should be strongly suspected in any child with persistent fever and organomegaly. As any infection can trigger HLH, an appropriate and thorough search for infective causes should be done in such cases.

Our case series highlights the need to search for all possible infective causes thoroughly in HLH, including all prevalent tropical infections and SARS-CoV-2 infection. Treatment of the primary etiology many times is sufficient and not all cases need immunosuppressive therapy.
